# The Emerging Role for Zinc in Depression and Psychosis

**DOI:** 10.3389/fphar.2017.00414

**Published:** 2017-06-30

**Authors:** Matthew A. Petrilli, Thorsten M. Kranz, Karine Kleinhaus, Peter Joe, Mara Getz, Porsha Johnson, Moses V. Chao, Dolores Malaspina

**Affiliations:** ^1^Creedmoor Psychiatric CenterQueens, NY, United States; ^2^Departments of Cell Biology, Physiology and Neuroscience, and Psychiatry, Skirball Institute of Biomolecular Medicine, New York UniversityNew York, NY, United States; ^3^Department of Psychiatry, New York University School of MedicineNew York, NY, United States

**Keywords:** zinc dysfunction, psychosis, depression, glutamate, NMDA

## Abstract

Zinc participation is essential for all physiological systems, including neural functioning, where it participates in a myriad of cellular processes. Converging clinical, molecular, and genetic discoveries illuminate key roles for zinc homeostasis in association with clinical depression and psychosis which are not yet well appreciated at the clinical interface. Intracellular deficiency may arise from low circulating zinc levels due to dietary insufficiency, or impaired absorption from aging or medical conditions, including alcoholism. A host of medications commonly administered to psychiatric patients, including anticonvulsants, oral medications for diabetes, hormones, antacids, anti-inflammatories and others also impact zinc absorption. Furthermore, inefficient genetic variants in zinc transporter molecules that transport the ion across cellular membranes impede its action even when circulating zinc concentrations is in the normal range. Well powered clinical studies have shown beneficial effects of supplemental zinc in depression and it important to pursue research using zinc as a potential therapeutic option for psychosis as well. Meta-analyses support the adjunctive use of zinc in major depression and a single study now supports zinc for psychotic symptoms. This manuscript reviews the biochemistry and bench top evidence on putative molecular mechanisms of zinc as a psychiatric treatment.

## Introduction

Zinc is an essential trace element required by all organisms for various biological processes. Its general actions are well reviewed, as briefly described in this paper and several excellent reviews (Marger et al., 2014; Nowak, 2015; Prakash et al., 2015), but a role for zinc homeostasis with respect to clinical depression and psychosis is not well appreciated by psychiatrists. Zinc is the second most abundant divalent cation after calcium and is a component in hundreds of enzymes and proteins. Playing a determinant role in over 300 biological processes, zinc is required for proper cellular function, including DNA replication, transcription, protein synthesis, maintenance of cell membranes, cellular transport, as well as endocrine, immunological and neuronal systems (Prasad, 1995; Nowak et al., 2003b). Dysregulation of zinc is associated with reduced immunological functioning, stunted tissue regeneration, growth retardation, gastrointestinal complaints, and ocular and sensory disturbances. Zinc insufficiency is also associated with neuropsychiatric manifestations that can present as altered behavior and cognition, reduced ability to learn, and depression (Nowak, 2001; Nowak et al., 2003a; Howland and Wang, 2008). The purpose of this review is to increase the appreciation of zinc with respect to psychiatric disorders.

The connection between zinc dysregulation and psychiatric illness is being continually clarified. Within the limbic system, zinc is predominately sequestered within glutamatergic neurons, typically acting as an inhibitory modulator at the NMDA glutamate receptor (Frederickson et al., 2000; Paz et al., 2008; Szewczyk et al., 2010). In addition to antagonism at NMDAR (*N*-methyl-d-aspartate receptor), and beyond the scope of this review, other zinc actions that may contribute to the prevention or presumed amelioration of depression include agonistic properties for AMPAR (α-amino-3-hydroxy-5-methyl-4-isoxazolepropionic acid receptor) and complex interactions with 5-HT1A receptors (agonism and antagonist and pre and postsynaptic). In addition, zinc is an agonist for GPR39 activation and for mTOR (mammalian target of rapamycin) (Szewczyk et al., 2015). Inhibitory actions at nAChR (nicotinic cholinergic receptor), GSK3β (glycogen synthase kinase 3beta) and NOS (nitric oxide synthase) are also pertinent to mechanisms of depression (Nowak, 2015).

## Zinc homeostasis and regulation

Zinc is an essential element obtained from dietary sources, particularly red meat, poultry, fish, and dairy. Tight regulation of zinc concentrations is essential as dietary intake of zinc varies as much as 15-fold. Its concentration is normally maintained by easy absorption in the digestive tract, but insufficiency can be related to dietary habits, aging, medical comorbidities (including alcoholism and H Pylori) and numerous common medications (including antacids, diuretics, anticonvulsants, anti-retrovirals, hormones, steroids, other anti-inflammatories, and numerous cardiovascular medications). Zinc is present in all body tissues, having higher concentrations within muscle and bone. The vast majority of zinc is protein bound and not in its free form with blood levels normally maintained between 9 and 17 μM.

Zinc is also tightly regulated in the brain, with higher levels in the amygdala, hippocampus and cortex, predominantly located within glutamatergic neurons known as “zinc enriched neurons” (ZEN) (Nowak et al., 2003b; Prakash et al., 2015). About 15% of all zinc in the CNS is in vesicular form within ZENs (Nowak, 2001). All ZENs are glutamatergic, although not all neurons that release glutamate contain zinc (Nowak et al., 2003b). With neuronal stimulation, zinc is released into the synapse and the cytosol concentration of zinc transiently reaches micromolar ranges, a level at which it physiologically regulates many synaptic processes.

## Zinc dysregulation and depression

There appears to be a correlation between zinc dysregulation in both neurological and psychiatric illnesses such as Parkinson's disease, Alzheimer's, Amyotrophic Lateral Sclerosis, Down Syndrome, attention deficit disorder hyperactivity and depression (Grabrucker et al., 2011). The association to depression may account for its largest psychiatric impact. Major depressive disorder causes significant morbidity and mortality affecting approximately 350 million people worldwide (WHO, 2012; Vashum et al., 2014). The World Health Organization predicts that by 2020 major depressive disorder will be the second leading cause of morbidity and mortality after ischemic heart disease (Mathews et al., 2012; WHO, 2012; Sowa-Kućma et al., 2013). Associated with decreased quality of life, depression results in over one million deaths by suicide per year. Multiple studies demonstrate reduced serum zinc levels in depressed individuals compared to healthy controls with a meta-analysis demonstrating depressive symptomatology at zinc serum levels of 1.8 μM or less (Swardfager et al., 2013). Consistent with dose effects and causality, an inverse relationship was observed between lower zinc levels and higher Hamilton Depression Rating Scale scores (Maes et al., 1994; Liuzzi and Cousins, 2004). Clinical studies show lower serum zinc levels in groups of cases with major depression (McLoughlin and Hodge, 1990; Maes et al., 1994; Siwek et al., 2010; Swardfager et al., 2013). Several randomized controlled trials support the effectiveness of zinc as adjunctive therapy for improving mood in both depressed and healthy individuals (Nowak et al., 2003a; Siwek et al., 2009; Sawada and Yokoi, 2010; Lai et al., 2012; Ranjbar et al., 2014; Solati et al., 2015). Zinc supplementation also improved mood in cases with treatment-resistant depression in several studies (Nowak et al., 2003a; Siwek et al., 2009). Recent meta-analyses demonstrated lower serum zinc levels in groups of cases with depression compared to controls, with significant inverse associations between depression severity scores and serum zinc levels and also demonstrating larger effect sizes in hospitalized cases (Swardfager et al., 2013, 2015). Studies on zinc deficiency and zinc supplementation in relation to depression are summarized in Table 1.

**Table 1:** Studies relating depression to zinc levels.

**Author, Year**	**Model**	**Subjects**	**Measure**	**Results**
Doboszewska et al., 2015	Zinc deficiency, depression	Rodent	FST, Western blot	Normalization of behavior, serum zinc level, and hippocampal GluN1, GluN2A, GluN2B, p-CREB after fluoxetine administration in zinc-deficient mice
Młyniec et al., 2014b	Zinc deficiency, depression	Rodents + suicide victims	FST, Western blot	Increased immobility time; downregulation of GPR39 receptor, CREB/BDNF/TrkB in zinc-deficient rodents and suicide victims
Młyniec et al., 2014a	Depression	Rodent	FST, TST	Increased immobility time in GPR39 knockout mice and downregulation of hippocampal CREB/BDNF
Młyniec et al., 2015	Depression	Rodent	FST	Antidepressant effect only with NMDA antagonists (but not monoamine-based antidepressants) in GPR39 knockout
Nowak et al., 2003a	RCT, zinc supplementation	Unipolar depression on TCA or SSRI (*n* = 14)	HADRS	Reduced depression scores after 6 and 12 weeks of zinc supplementation compared to placebo
Nowak et al., 2003b	Suicide	Suicide victims (*n* = 10)	Radioligand binding assay	Reduced potency (26% decrease) of zinc to inhibit MK-801 (an NMDA antagonist) binding to NMDA receptors in hippocampal tissue of suicide victims
Ranjbar et al., 2014	RCT, zinc supplementation	Major depression on antidepressant (*n* = 37)	HDRS; serum IL-6, TNF-alpha, BDNF	Significantly reduced HDRS after 12 weeks zinc supplementation, but no change in inflammatory cytokines or BDNF
Sawada and Yokoi, 2010	RCT, zinc supplementation	Healthy premenopausal women (*n* = 30)	Anger-hostility and depression-dejection scores in Profile of Mood States (POMS)	Improvement in anger-hostility and depression-dejectino scores after 10 weeks zinc supplementation + MV vs. MV alone
Siwek et al., 2009, 2010	RCT, zinc supplementation	Unipolar depression on imipramine therapy (*n* = 60)	HDRS, BDI, CGI, MADRS	Reduced depression scores only in treatment-resistant patients, but not in antidepressant responders
Solati et al., 2015	RCT, zinc supplementation	Obese or overweight, regardless of depression status	BDI; serum zinc, BDNF	Higher serum zinc and BDNF and greater reduction in BDI score in zinc-supplemented group; BDI change only in depressed subgroup; negative correlation between serum BDNF and depression; positive correlation between serum BDNF and zinc levels at baseline
Sowa-Kućma et al., 2013	Suicide	Suicide victims (*n* = 17)	Radioligand binding assay	Reduced potency (29% decrease) of zinc to inhibit MK-801 (an NMDA antagonist) binding to NMDA receptors in hippocampal tissue of suicide victims
Swardfager et al., 2013	Meta-analysis, depression	Human, Depressed vs. control	Serum zinc levels	Zinc concentrations approximately 1.85 umol/L lower in depressed subjects than control subjects
Szewczyk et al., 2009	Zinc supplementation	Rodent	FST	Decreased immobility time with zinc; effect diminished by NMDA administration and AMPAR antagonist
Szewczyk et al., 2010	Zinc supplementation	Rodent	FST	Decreased immobility time with combined zinc and citalopram or fluoxetine at sub-effective doses; effect blocked by ritanserin and WAY 1006335
Vashum et al., 2014	Dietary zinc, depression risk	Two prospective Australian cohorts (*n* = 2092 men and women, *n* = 9738 women)	Centre for Epidemiological Studies Depression Scale (CESD)	Dietary zinc associated with lower incidence of depression in men and women 50 years and older

The relationship between zinc and depression may be linked to its action on brain-derived neurotropic factor (BDNF), a growth factor promoting neurogenesis and differentiation. The hippocampus is a site of life long neurogenesis, with decreased BDNF expression and diminished neuro/synaptogenesis accompanying episodes of major depression. Rodents fed a diet deficient in zinc demonstrated reduced zinc levels in the hippocampal vesicles, an area of the brain that normally has higher concentrations, with accompanying decreases in progenitor cells and immature neurons. The contrary was observed with zinc-enriched diets, with an increase in progenitor cells (Nowak et al., 2003a; Suh et al., 2009). Zinc interacts with BDNF levels and its deficiency thereby decreases neurogenesis and depressive symptoms ensue. An inverse correlation was observed between serum BDNF levels and depression severity in one clinical trial (Ranjbar et al., 2014). The exact relationship between BDNF and zinc is being elucidated, although a possible role for zinc in synaptogenesis includes its role in transactivating TrkB, a crucial neurotrophic factor (Huang et al., 2008). Zinc's activation of TrkB is independent of BDNF activation and produces hippocampal mossy fiber potentiation. Further studies with mice suggest that not only zinc is required for mossy fiber potentiation, it can also inhibit it postsynaptically. This suggests that zinc may be required as a dual control to maintain homeostasis (Pan et al., 2011). Zinc thus regulates synaptic plasticity, aiding in neurogenesis and preventing pathological states.

Further alluding of zinc's complex role with BDNF, is zinc's interaction with GPR39 receptors. Zinc acts as a natural ligand which stimulates GPR39 resulting activation of G-protein signal transduction pathways. In rodent models, GPR39 knockout mice resulted in decreased levels of BDNF in the hippocampus along with increased immobility time in both the forced swim test (FST) and tail suspension test (TST) which is analogous to the depressive phenotype (Młyniec et al., 2014a). The GPR39 receptor likely serves as a crucial link in the interaction between zinc and the serotonergic system, necessary for the activity of antidepressants targeting the serotonin pathway (Doboszewska et al., 2017).

## Zinc deficiency in relation to psychotic disorders

Schizophrenia is a disabling syndrome of psychosis and impaired functioning with both neurodevelopmental and degenerative pathologies that affects nearly 21 million individuals worldwide (WHO, 2016). The condition has neurodevelopmental underpinnings and prenatal zinc deficiency may also be relevant, whether a consequence of maternal zinc insufficiency or fetal gene variants that impact the movement of zinc across cellular membranes. Prenatal zinc deficiency produces decreased brain volume in rodent models, consistent with impaired cell proliferation and retarded neuronal maturation (Dvergsten et al., 1983, 1984a,b; Takeda and Tamano, 2009). This is relevant to the risk of schizophrenia, as a 30–50% reduction in brain zinc content is demonstrated for early onset cases compared to control samples in postmortem samples (Kimura and Kumura, 1965; McLardy, 1973). The expression of the schizophrenia phenotype may reflect interactions of prenatal zinc deficiency with other risk genes, and/or ongoing deficiency following birth. Studies on zinc deficiency and zinc supplementation in relation to psychosis are summarized in Table 2 and are discussed in the following sections.

**Table 2:** Studies relating psychosis to zinc levels.

**Author, Year**	**Model**	**Subjects**	**Measure**	**Results**
Czerniak and Haim, 1971	Zinc supplementation	80 mice, 80 rats	Injection of 5 and 15 microcuries of zinc chloride Zn 65	Phenothiazine compounds increase the total brain zinc uptake in all animals
Holcomb et al., 2005	Human drug administration	Normal volunteers (*n* = 13); Schizophrenic volunteers (*n* = 10)	Drug-induced NMDAR antagonist ketamine (0.3 mg/kg)	Schizophrenic volunteers showed greater relative blood flow increases in the anterior cingulate and correlated with changes in psychosis ratings; ketamine-induced inhibition and increased glutamate release may cause the distorted thoughts and diminished cognitive abilities elicited by NMDAR blockade
Kimura and Kumura, 1965	Human zinc levels	Cases with typical schizophrenia (*n* = 10) and cases of various cerebral diseases (*n* = 10)	Polarogram	Zinc level in brain regions is significantly lower in the schizophrenia group than other diseases
Mortazavi et al., 2015	Double-blind randomized placebo-controlled trial	Schizophrenia inpatients (*n* = 30) receiving 6 mg/day risperidone	Capsules of adjunct Zn sulfate (each containing 50 mg elemental Zn) three times a day	Psychotic symptoms and aggression risk decreased for both groups; higher improvement for Zn sulfate receiving group than placebos
McLardy, 1973				30% deficit of brain Zn2+ content in individuals with early onset schizophrenia
Tokdemir et al., 2003	Human zinc levels	88 schizophrenic patients from Elazig Mental Hospital (*n* = 44 w/criminal record, *n* = 44 no crimincal record	5 mL (IV) heparin blood draw for plasma zinc concentration	Mean plasma zinc values significantly lower in criminal subjects when compared to non-criminal subjects; 68 ± 1.55 microg/dL mean in the criminal subjects and 81 ± 2.73 microg/dL mean in the non-criminal subjects (*p* = 0.001)
Walsh et al., 1997	Human zinc levels	All male patients between the ages of 3–20 years who made a first visit to the outpatient Pfeiffer Treatment Center in Naperville, Ill., during a 2-month period (*n* = 135 assaultive young males, *n* = 18 controls w/no history of assaultive behavior	Blood samples using atomic absorption methods	Depressed plasma zinc in blood samples collected from violence-prone individuals; median Cu/Zn ratio for the assaultive subjects was 1.40 compared to 1.02 for controls, a statistically significant difference (*t* = 5.94; *p* < 0.0.01)

## Zip proteins and the SLC39A13 mutation

Abnormalities in intracellular zinc may occur even when serum levels are normal if there is dysfunction in the molecules that move zinc across membranes. The two major families of zinc transporters include the SLC family (ZnT) and the SLC39 family (ZIP). The family of ZIP proteins includes 14 mammalian members responsible for transporting zinc into the cytoplasm, whereas the ten mammalian ZnT transport zinc into the extracellular space (Ilouz et al., 2002; Jeong and Eide, 2013).

The SLC39 solute carrier genes are of particular interest, as recent research identified point mutations in association with severe mental illness (Ilouz et al., 2002; Fukada et al., 2008; Kranz et al., 2015). In one study, a disruptive *de novo* mutation was identified in the zinc transport gene SLC39A13 in a sporadic schizophrenia case compared to healthy parents. A subsequent New York Study identified 4 other rare/novel disruptive variants in this same gene from among 48 unrelated cases (Fukada et al., 2008; Kranz et al., 2015). This gene, also known as ZIP13, is located on chromosome 11 (Kranz et al., 2016). It is responsible for the influx of zinc into the cytoplasm from the extracellular space and/or the efflux of zinc from intracellular organelles. The gene is widespread throughout the body and found in high concentrations within the Golgi apparatus (Kranz et al., 2016).

It is relevant that assaultive behavior in humans (Tokdemir et al., 2003; Młyniec et al., 2014b) and greater aggression in rodents are observed with zinc deficiency. In 1997 Walsh and colleagues demonstrated significantly lower zinc serum values in criminals with schizophrenia vs. non-criminal subjects (Walsh et al., 1997). The neurobiology associated with the SLC39A13 mutation was further characterized through a magnetic resonance imaging (MRI) approach, which demonstrated significantly reduced neuronal concentrations in the rostral anterior cingulate cortex, which was significantly associated in the imaged sample of cases with reduced verbal intelligence and stable (trait) negative symptoms (Malaspina et al., 2016).

As to mechanisms, it is notable that the SLC39A13 gene plays an important role in BMP/TGF-β signaling pathways, which has crucial roles in development and a host of cellular processes. With respect to schizophrenia, the pathway regulates oligodendrocyte maturation and differentiation (McKinnon et al., 1993; Palazuelos et al., 2014). It also participates in the development of connective tissues (Ilouz et al., 2002; Marger et al., 2014). One particular SLC39A13 mutation is linked to spondylocheiro dysplastic-Ehlers–Danlos syndrome, a condition which includes spine and hand dysplasia and can also entail significant psychopathology, including schizophrenia, in association with joint hypermobility (Sinibaldi et al., 2015; Malaspina et al., 2016). Maintaining zinc levels within a narrow range is also essential for glutamatergic function; another important mechanism as the receptor interacts with zinc. Rodents with zinc deficiency demonstrate alterations in glutamatergic receptor function with reduced neuroplasticity and neurogenesis. This interaction is dysregulated in psychiatric disease states such as schizophrenia and depression (Nowak, 2001; Szewczyk et al., 2010; Prakash et al., 2015). A brief review of the relevant NMDA receptor anatomy and physiology is presented below to illustrate zinc's actions.

While this review emphasizes the role of the SLC39A13 gene, recent studies have shed light on additional zinc transport genes linked to schizophrenia. SLC39A12 was previously identified as a candidate (Bly, 2006), and increased cortical expression of this gene and increased zinc uptake have been demonstrated in post-mortem brain tissue (Scarr et al., 2016). Furthermore, these changes are specific to schizophrenia and do not occur in subjects with mood disorders. SLC39A8 is also implicated in schizophrenia, and genome-wide association studies (GWAS) now reveal shared genetic influences of the SLC39A8 gene on both schizophrenia and inflammatory bowel disease (Pickrell et al., 2016). Variants of SLC39A8 are associated with a shift in gut microbiome composition, T cell immunity, lipid levels, blood pressure, and obesity, highlighting the relationship between schizophrenia, inflammation, and metabolic dysregulation (Marger et al., 2014; Li et al., 2016). Lastly, outside of the SLC39 family, alleles of the SLC30A3 gene have also been shown to confer risk of schizophrenia on female but not male individuals (Perez-Becerril et al., 2016). These studies demonstrate the expanding role of zinc homeostasis on schizophrenia and other disorders.

## NMDA receptor anatomy and physiology

**Figure 1:**
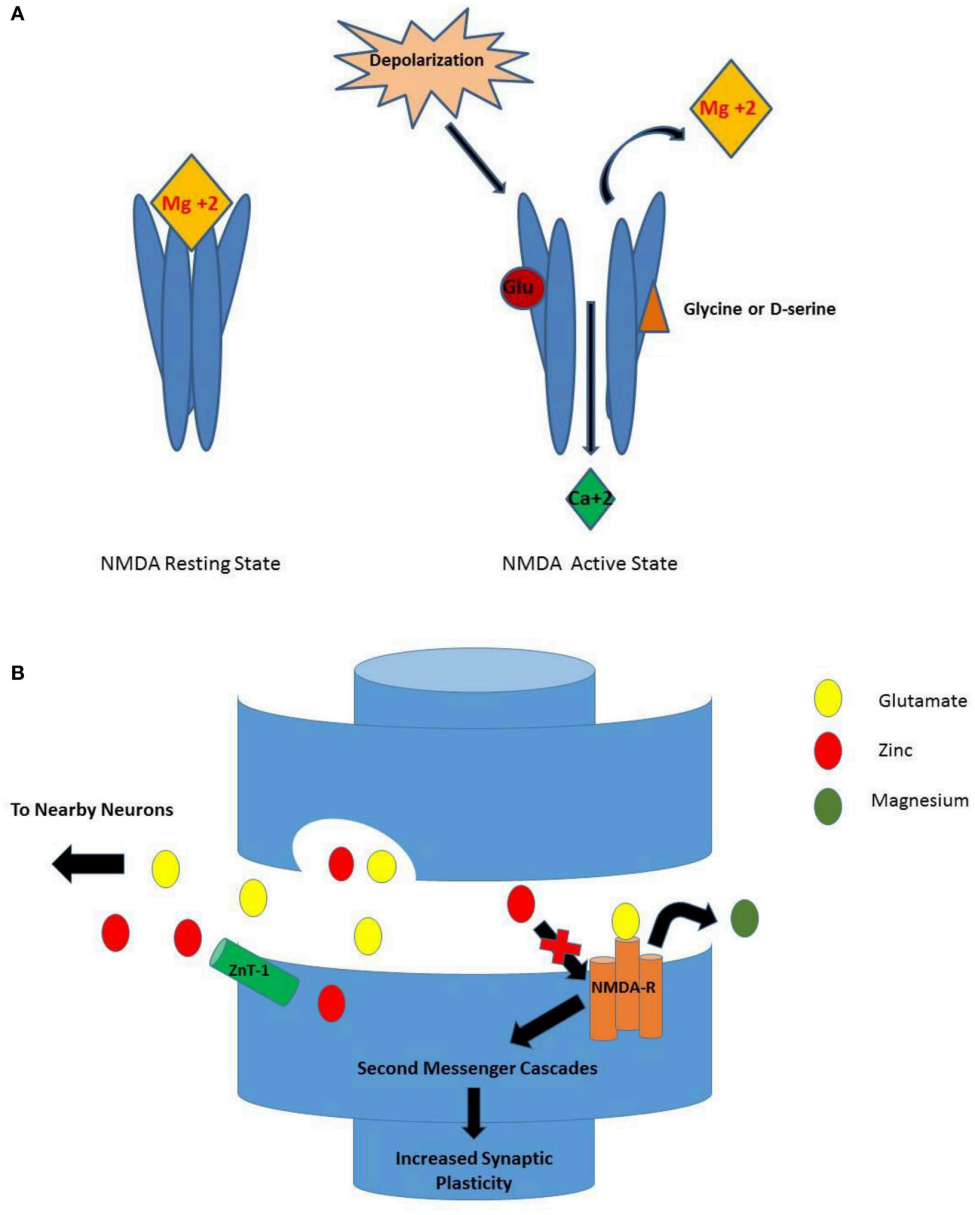
**(A)** NMDA Receptor Activation. While in the resting state, Mg^+2^ blocks the Ca^+2^ ionic pore. For a neuron to become depolarized, both glutamate and glycine (or D-serine) must bind to their respective sites for removal of the Mg^+2^ ion and permit entry of Ca^+2^ through the pore. **(B)** Activation of NMDA-R as Seen in Synapse. When depolarization occurs, glutamate is released from the presynaptic terminal and binds to the NMDA receptor. Once serine binds, the Mg^+2^ ion is released from the ionic pore allowing Ca^+2^ to enter. The result is a variety of second messenger cascades that result in increased synaptic plasticity. Conversely, when zinc is present in the synapse, the activation of the NMDA receptor is inhibited (Walsh et al., 1997).

The N-methyl-D-aspartate (NMDA) receptor is a member of the ligand-gated ion channel family of receptors, which includes the AMPA and kainate glutamate receptors. The NMDA receptor is a multi-domained ion channel that is principally permeable to calcium (Ca^+2^), and to a lesser extent, to sodium (Na^+^) and potassium (K^+^) (Nowak, 2001). Although there is vast molecular diversity of NMDA receptors across anatomical locations and physiologic conditions, the core anatomy includes a heterotetramer of two glycine-binding (NR1) subunits and two glutamate-binding (NR2) subunits (Figure 1) (Sowa-Kućma et al., 2013). NMDA receptors tend to be post-synaptic and to collaboratively modulate the excitatory post-synaptic transmission stimulated by glutamate, also by allosteric modulation by glycine, polyamines and zinc (Nowak, 2001).

In order for the NMDA receptor to function, glutamate's co-transmitter glycine must be present (Sowa-Kućma et al., 2013). Glycine is provided by neighboring glia and released into the synapse for readily available binding (Nowak, 2001; Sowa-Kućma et al., 2013). Closely related to glycine and synthesized by serine racemase, D-serine is a potent allosteric modulator that can be used interchangeably at the glycine allosteric site. D-serine co-exists with glycine at the human synapse; its actions at the glycine allosteric site are beyond the scope of this review (Hashimoto, 2014; Balu, 2016). When magnesium ion (Mg^+2^) is situated at the entrance of the calcium channel it forms a plug (Mathews et al., 2012) which blocks the NMDA receptor. The necessary “perfect storm” for the mechanical changes that open the channel entail glutamate binding and then the binding of the co-transmitter glycine (or D-serine). These events displace Mg^+2^ so calcium can enter and depolarize the neuron. This leads to signal cascades and in some pathways producing long-term potentiation and increased synaptic plasticity (Figure 1; Sowa-Kućma et al., 2013).

Predominately in the limbic regions, zinc is highly concentrated in hippocampal mossy fiber vesicles and the axons of dentate granule cells (Marger et al., 2014). While glycine potentiates NMDA receptor's activity leading to calcium entry and subsequent neural depolarization, zinc inhibits this action (Nowak, 2001; Nowak et al., 2003b). It is released concurrently with glutamate (by ZENs) and rapidly reaches micromolar levels which are necessary for synaptic modulation (Marger et al., 2014). The binding dynamics of zinc to the glutamate-binding NR2 subunit of the NMDA receptor vary across the glutamate binding isoforms. NR2A, which is synaptic, has a high sensitivity to extracellular zinc and requires only nanomolar concentrations to produce inhibition in a voltage-independent fashion (Low et al., 2000; Marger et al., 2014). By contrast, the extrasynaptic NR2B subunit binds zinc at a 100-fold lesser affinity than NR2A to produce voltage dependent inhibition (Sowa-Kućma et al., 2013) consistent with zinc entering the ionic pore. The mechanism of sensitivity to zinc by NR2C and NR2D is less clear, but multiple other processes also entail synaptic modulation by zinc in the micromolar range (Marger et al., 2014).

In addition to modulating NMDA receptor activation via allosteric receptor binding (Howland and Wang, 2008; Marger et al., 2014; Prakash et al., 2015), zinc also stimulates the release of the inhibitory neurotransmitter GABA from interneurons for presynaptic inhibition of glutamate release (Howland and Wang, 2008). With less glutamate in the synapse, glutamate binding at the NMDA receptor is consequently reduced (Salari et al., 2015). Furthermore, the dehydrogenase and decarboxylase enzymes that catabolize glutamate have reduced activity in the presence of zinc (Prakash et al., 2015). Lastly, zinc inhibits the group I metabotropic glutamate receptors (mGluR), diminishing Ca^+2^ release from internal neuron stores. Ca^+2^ enhances the activity of the NMDA receptor, so zincs role to decrease the Ca^+2^ availability further reduces NMDA receptor functionality (Howland and Wang, 2008; Salari et al., 2015).

The complex interactions between zinc and the glutamatergic system in normal physiological conditions produce a tightly controlled, activity-dependent homeostasis. When these interactions are perturbed, deleterious effects such as excitotoxicity can occur (Howland and Wang, 2008; Marger et al., 2014; Prakash et al., 2015; Salari et al., 2015), which is a risk pathway for a multitude neuropsychiatric disorders. Excitotoxicity describes the pathology of neuronal death or damage resulting from excessive transmission by glutamate and some similar substances. Excitotoxins include NMDA and kainic acid, which bind to the NMDA and AMPA receptors, as well as pathologically high levels of glutamate. These toxins cause excitotoxicity by allowing high levels of Ca2+ to enter the cell which in turn activates a number of toxic enzymes, including phospholipases, endonucleases, and proteases such as calpain that damage the cellular cytoskeleton, membranes, and genetic material.

## Zinc modulates NMDA receptor activity

**Figure 2:**
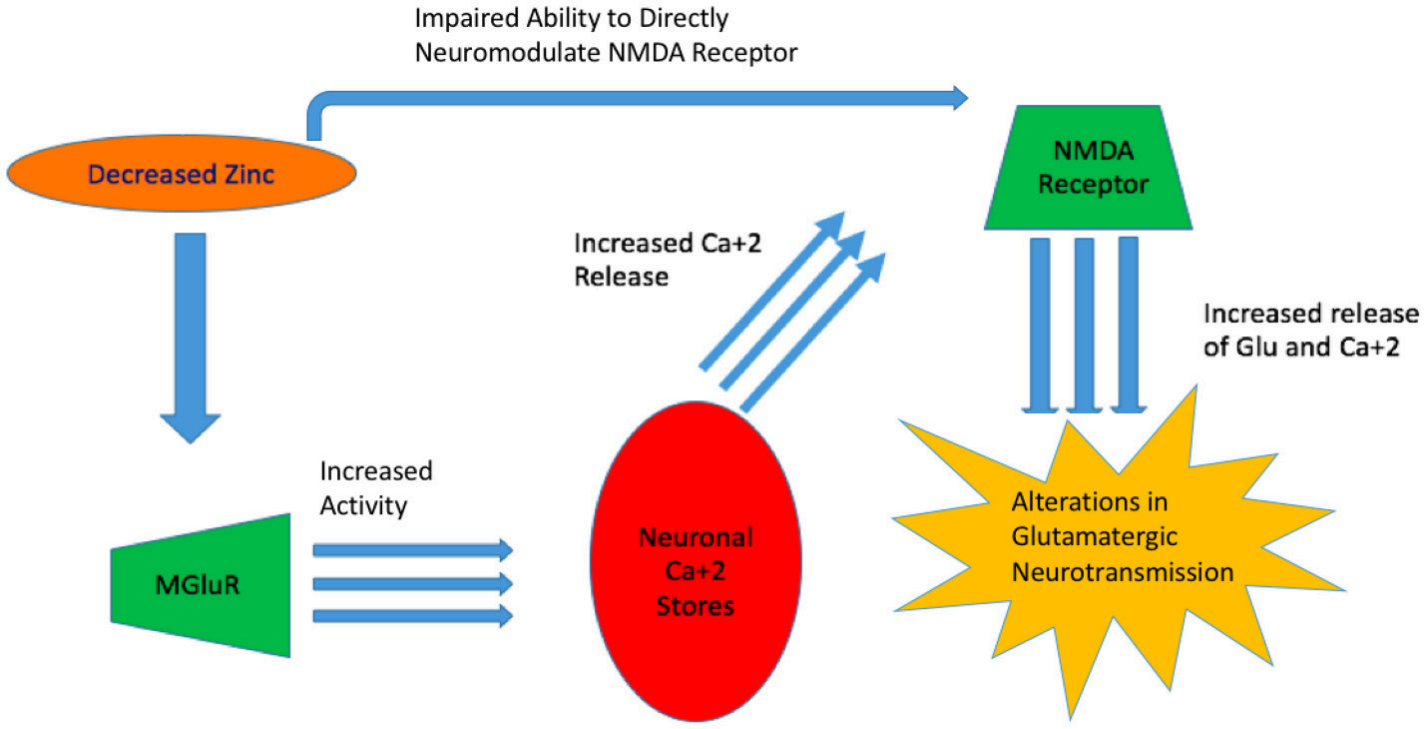
Zinc's Impaired Ability to Act as a Neuromodulator. With decreased zinc available, MGluR increases in activity and increased neuronal stores of Ca^+2^ are released ^2^ which leads to alterations in glutamatergic neurotransmission. Concurrently, decreased available zinc, impairs its ability to directly act on the NMDA receptor and resulting in overactivation.

Glutamate homeostasis and neurotransmission are dysregulated in many mental conditions (Paz et al., 2008; Szewczyk et al., 2010). As zinc is a modulator of NMDA receptor activity together with Mg^2+^ and H^+^, significant deficiency can increase the propensity of NMDA receptor activation (Low et al., 2000). Notably, the ability of zinc to alter receptor activation sensitivity depends on the NMDA receptor subunit composition. In one study, HEK293 cells were used for the overexpression of NR1/NR2A and NR1/NR2B subunit complexes and electrophysiologically assessed. The experiments revealed that NR1/NR2A mediated current responses were more sensitive to extracellular zinc than those mediated by NR1/NR2B (Chen et al., 1997). This effect is explained by a higher affinity of zinc to its binding site in the NR1/NR2A complex (Paoletti et al., 1997; Rachline et al., 2005) (Figure 2).

## Glutamatergic hyperactivity and zinc dysregulation in depression

Zinc and other antagonists of the NMDA receptor show antidepressant-like effects (Autry et al., 2011), attributed to the inhibition of NMDA-sensitive glutamate-gated channels, and include preclinical and clinical studies (Szewczyk et al., 2008). Zinc's negation of depressive features was demonstrated in rodents using the forced swim test (Szewczyk et al., 2010), wherein zinc pretreatment was related to longer periods of escape behaviors before immobility (i.e., less depressive-like behavior). Zinc administration in rodents also reduced the number of NMDA receptor complexes, suggesting downregulation (Cichy et al., 2009; Szewczyk et al., 2009, 2010). Conversely, a zinc-deficient diet induces upregulation of NMDA receptor complexes, but these levels normalize following antidepressant treatment, along with reversal of depression-like behavior in mice (Doboszewska et al., 2015).

Depressive-like effects may be consequences of altered NMDA receptor subunit concentrations (Tokita et al., 2012; Sowa-Kućma et al., 2013). In a 2008 post-mortem study of suicide victims, Sowa-Kucma et al. observed a reduced affinity of zinc to interact with hippocampal NMDA receptor subunits compared to controls, even without changes in zinc concentrations. Concurrently, NR2A subunits were significantly elevated while NR2B subunits appeared to be decreased (Sowa-Kućma et al., 2013), consistent with hypersensitivity of the NMDA receptor and compensatory upregulation of NR2A subunits to mitigate zinc actions at the receptor. Victims of suicide diagnosed with depression also showed reduced potency of zinc to inhibit the hippocampal NMDA receptor without zinc deficiency (Nowak et al., 2003b).

Research examining serotonin receptor reuptake inhibitor (SSRI) effects on the NMDA receptor uphold the hypothesis of alterations in the receptor complex during major depressive disorder. Chronic antidepressant treatment decreases the affinity for glycine, reduces glycine-displaceable glutamate sites (Nowak, 2001) and NMDA receptor complex function in the hippocampus (Paul et al., 1994; Nowak, 2001). Chronic antidepressant treatment was similarly observed to change the mRNA expression of genes that encode for the NMDA receptor subunits, resulting in decreased expression and/or decreased functionality of the NMDA receptor, helping to protect against glutamate mediated excitotoxicity (Szewczyk et al., 2010). Not only does chronic antidepressant treatment lead to changes within the human NMDA receptor itself, but levels of zinc, previously decreased, appear to normalize (Wójcik et al., 2006; Siwek et al., 2010; Prakash et al., 2015). A statistically significant 20% increase in hippocampal zinc was observed after 14 days of citalopram administration to rodents (Doboszewska et al., 2015) with similar effects from electroshock in rodents (Nowak and Schlegel-Zawadzka, 1999; Vaidya et al., 1999; Nowak, 2001). As the hippocampus is a major site of synapto/neurogenesis, which is greatly reduced in major depressive disorder (Swardfager et al., 2013; Prakash et al., 2015), the increased hippocampal zinc concentration after successful antidepressant treatment supports its role in neurogenesis as well as in neuroprotection.

The role of serotonergic transmission in depression is well described and has led to the production of many pharmaceuticals for depression treatment. However, its interaction with glutamate and specifically with the NMDA receptor is another putative pathway for treating depression (Szewczyk et al., 2010). Serotonergic receptors (primarily 5-HT1A) and zinc interact, as observed in the rodent forced swim test (Jeong and Eide, 2013; Nowak, 2015) by complete abatement of the antidepressant effects of zinc in the presence of a 5-HT1A antagonist (Rachline et al., 2005; Satała et al., 2016). The inverse is also exhibited in rodents when zinc was co-administered with SSRIs. The interaction appeared to be synergistic with increases in motility time in the forced swim test. Zinc may serve as a possible allosteric modulator of 5-HT1A receptors, capable of inhibiting both agonist and antagonist binding at relevant concentrations in the synapse (Prakash et al., 2015). Another way that zinc may interact with the serotonergic system is via the previously mentioned GPR39 receptor. In GPR39 knockout mice, only NMDA antagonists, but not monoamine-based antidepressants, have antidepressant effect, thus establishing a connection between zinc, serotonin, and BDNF expression (Młyniec et al., 2015). This research further alludes to serotonin and zinc's interaction.

## Zinc and inflammation

**Figure 3:**
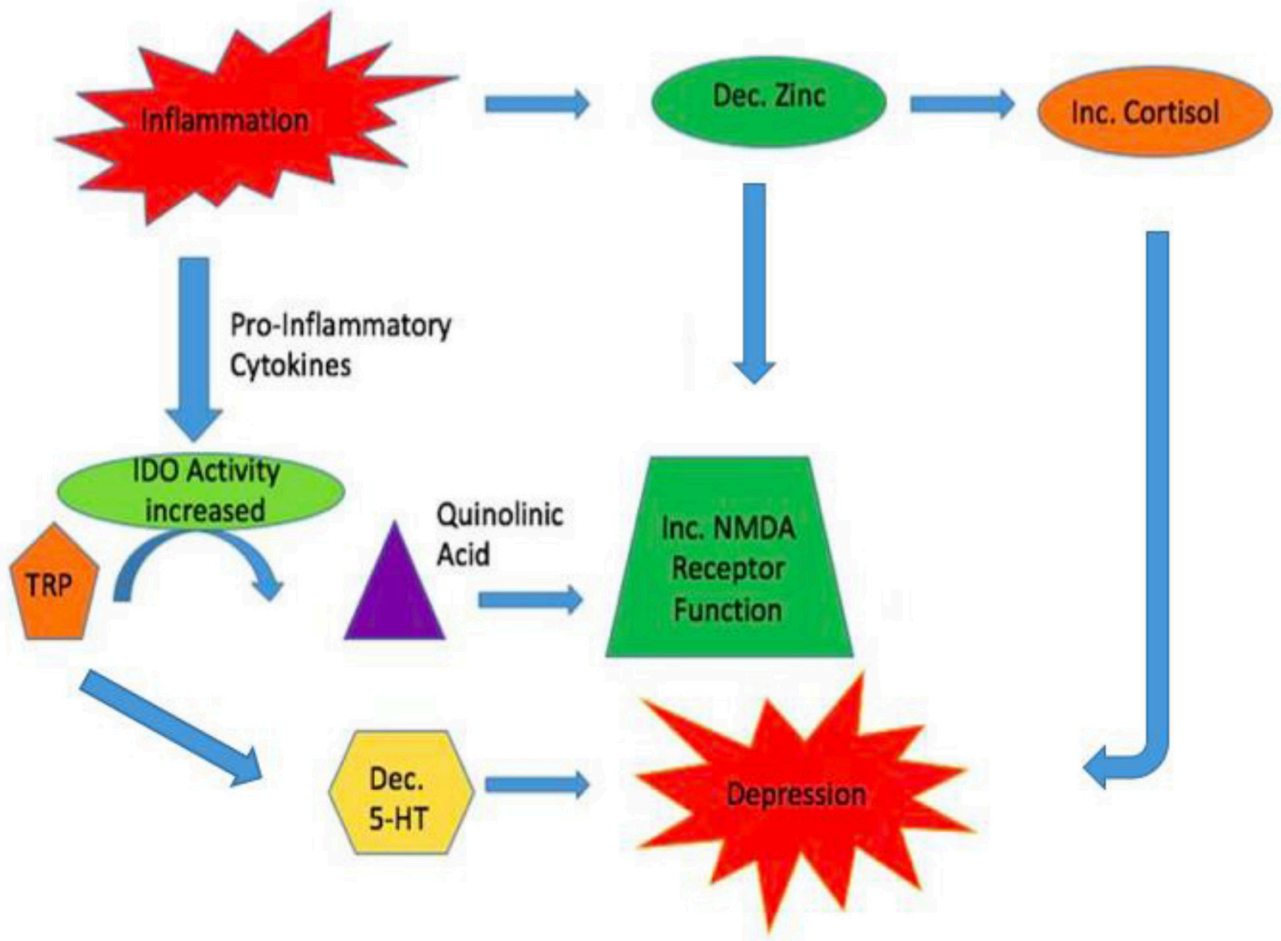
Inflammation may link the Glutamatergic and Serotonergic Systems. During times of inflammation zinc reserves are decreased. One result is the activation of the HPA axis with increases in glucocorticoids. This can result in symptoms of depression. Simultaneously, pro-inflammatory cytokines increase the enzyme IDO resulting in increased turnover of tryptophan to quinolinic acid, a NMDA receptor agonist. This leads to increased release of glutamate and Ca^+2^ increasing the chances for causing neuronal harm. Due to tryptophan being actively converted to quinolinic acid, less tryptophan is being diverted to produce serotonin which can add to symptoms of depression caused by increased glucocorticoids.

The inflammatory system is another probable link between the glutamatergic and serotonergic systems in major depressive disorder, as zinc levels are decreased by stress and inflammation (Prakash et al., 2015). Numerous literature has already demonstrated the increase in glucorticoids from HPA-axis dysregulation resulting in symptoms of depression. Decreased zinc within the hippocampus may activate the HPA-axis (Nowak, 2001). Concurrently, the enzyme indoleamine 2,3-dioxygenase (IDO) is stimulated by pro-inflammatory cytokines. It metabolizes tryptophan into quinolinic acid, a NMDA receptor agonist. It is hypothesized that the increase in IDO activity results in a decrease in available tryptophan for 5-HT synthesis, thus compounding the possible depressive symptoms that have been elicited from HPA-axis dysregulation (Liuzzi and Cousins, 2004). The increase in quinolinic acid results in excess NMDA receptor activity subsequently causing excess glutamate release and neurotoxicity (Prakash et al., 2015). With zinc already decreased in the pro-inflammatory state, it is unable to inhibit the NMDA receptor effectively and hyperactivity ensues, resulting in potential deleterious consequences (Figure 3).

## Glutamatergic hyperactivity in relation to zinc dysregulation in psychotic disorders

NMDA hypofunction was first implicated for psychosis when individuals ingesting PCP, an NMDA antagonist, exhibited thought disorganization, auditory hallucinations, emotional blunting, and cognitive disturbances. Psychotic symptoms were also observed in healthy individuals receiving sub-anesthetic doses of ketamine, an NMDA antagonist. Acute administration of ketamine was shown to trigger pyramidal neuronal excitation in the prefrontal cortex and increased metabolic activity (Holcomb et al., 2005; Wójcik et al., 2006). NMDA antagonists may block excitatory inputs to GABAergic interneurons, preventing the down-regulation of prefrontal glutamatergic neurons. By triggering signal transduction pathways that potentiate glutamatergic excitability (Paz et al., 2008), both psychosis and cognitive deficits may be elicited.

Some literature suggests that psychosis in schizophrenia is related to inflammation of brain tissue (Prakash et al., 2015). As described, inflammation leads to a decrease in zinc levels, resulting in increased NMDA receptor activity and ultimately to excitotoxicity as high levels of calcium enter the cell and activate enzymes that damage subcellular structures. Lower zinc levels in persons with schizophrenia may be a consequence of inflammation and/or genetic defects in molecules that maintain zinc homeostasis or nutritional deficiencies or malabsorption. Each can produce NMDA hyperactivity and possibly psychotic symptomatology. Modulating glutamatergic transmission within the neural networks that converge in the prefrontal cortex using pharmaceuticals that act on the NMDA receptor and/or zinc supplementation may remediate hyperglutamatergic states and possibly reduce psychotic symptoms (Paz et al., 2008).

All typical antipsychotics bind dopamine-2 (D2) receptors at efficacies that are related to antipsychotic symptom reductions. However, it may be D1 receptor antagonists that can diminish glutamatergic hyperactivity by reducing NMDA receptor activity (Paz et al., 2008). Strong D2 antagonists may have minimal effects at modulating glutamatergic activity. Interestingly, the antipsychotic clozapine, which is used for treatment refractory psychosis, has a higher affinity for the D1 than D2 receptors, possibly exerting a stronger effect on NMDA receptor hyperactivity in the prefrontal cortex (Paz et al., 2008). An inference is that the more robust response from clozapine may entail normalization of prefrontal glutamatergic activity. However, the interaction between D1 blockade and NMDA receptor downregulation is not direct, and not yet well described in terms of signal transduction. Direct modulation of hyperactive NMDA receptors by zinc inhibition may target mircocircuitry that is not accessible to the conventional D2 receptor antagonists (Paz et al., 2008).

Zinc supplementation may be a useful person-specific intervention. A double-blind, placebo-controlled schizophrenia study showed that 220 mg of zinc sulfate TID, used as an adjuvant to 6 mg/day of risperidone, produced a statistically significant improvement of positive and negative symptomatology and reduced aggressive behavior (Mortazavi et al., 2015). These results correlate with the aforementioned hypothesis that increasing zinc through dietary supplementation may be a route to inhibiting NMDA receptors, decreasing glutamate mediated excitotoxicity and thus normalizing glutamatergic transmission in the PFC. Perhaps a further study examining only schizophrenic individuals exhibiting the SLC39A13 mutation or reduced serum zinc levels would reveal a more robust response with zinc supplementation. As the psychoses are heterogeneous, it is unlikely that zinc supplementation would be useful for all cases.

## Future directions

Research from the last two decades suggests a robust amount of evidence connecting zinc dysregulation and deficiency to a multitude of neuropsychiatric conditions. Although there is only a limited amount of data regarding the efficacy of zinc as a treatment modality in psychiatric disorders, it seems quite reasonable, given the amount of substantiated data demonstrating the relevance of zinc dysregulation in psychiatric disorders to further pursue zinc as a potential therapeutic option in psychiatry. The complex relationship between the glutamatergic system and other neurotransmitter systems, including serotonin, dopamine, and inflammatory pathways, demonstrates the multi-faceted and more complex underpinnings of psychopathology exceeding earlier views of the dysregulation of a particular neurotransmitter. Although only one randomized, double blind controlled trial has been completed on zinc supplementation for treatment of schizophrenia, the Mortazavi et al. study remains promising. As the current understanding of schizophrenia becomes more refined, so will treatment modalities.

As pharmacotherapy is costly and comes with increased potential for adverse side-effects, zinc may serve as an adjuvant to help resolve symptoms of mental illness (Vashum et al., 2014; Mortazavi et al., 2015). Zinc is well tolerated with minimal side effects, other than occasional gastrointestinal disturbance. The most common method of zinc administration is oral dietary supplementation and comes in various compounds (i.e., Zinc oxide, zinc sulfate, zinc acetate, etc.) with various tolerability and absorption profiles. The rate of intestinal absorption of zinc has been observed to be increased when supplemented with vitamin B6 (Grabrucker et al., 2011). However, because zinc uptake into the CNS being an active transport process, it is hard to control the exact zinc level into the brain. There are limited studies which examine potential modalities to deliver zinc to specific brain areas, however certain compounds have shown to aid in increase zinc uptake in rodent models. It has been observed that phenothiazine derivatives which include chlorpromazine, thioridazine and perphenazine increased the absorption of zinc supplemented to rodents, however would possibly be counterintuitive to be adding first generation neuroleptics with high side-effect profiles to aid in CNS transport of zinc (Czerniak and Haim, 1971; Grabrucker et al., 2011).

As current research supports plausible roles for zinc in reducing both depressive and psychotic symptoms, zinc supplementation may reduce the amount of psychotropic medication required, leading to increased adherence, lower costs and more favorable outcomes. Due to the heterogeneity of mental illness, further study of certain subsets that would most benefit from zinc supplementation must be more clearly refined. Clearly more research is needed to elucidate the impact of zinc on neuropsychiatric conditions.

## Author contributions

All authors listed have made a substantial, direct and intellectual contribution to the work, and approved it for publication.

### Conflict of interest statement

The authors declare that the research was conducted in the absence of any commercial or financial relationships that could be construed as a potential conflict of interest.
